# Evaluation of Tibial Hemodynamic Response to Glucose Tolerance Test in Young Healthy Males and Females

**DOI:** 10.3390/nu15184062

**Published:** 2023-09-20

**Authors:** Si Chen, Shubo Wang, Shuqiao Ding, Chuan Zhang

**Affiliations:** 1 School of Physical Education and Sport, Central China Normal University, Wuhan 430079, China; disk1996@outlook.com (S.C.); dingshuqiao@ccnu.edu.cn (S.D.); 2 Globus Medical Inc., Audubon, PA 19403, USA; shubo.wang@globusmedical.com

**Keywords:** glucose metabolism, near-infrared spectroscopy, bone hemodynamics, bone oxidative metabolism

## Abstract

The relationship between glucose metabolism and bone health remains underexplored despite its clinical relevance. This study utilized the oral glucose tolerance test (OGTT) and near-infrared spectroscopy (NIRS) to probe gender-specific disparities in tibial hemodynamic responses among young healthy adults. Twenty-eight healthy participants (14 males) aged 18–28 years old were recruited for this study. After ingesting a 75 g glucose solution, tibial hemodynamic responses were captured using NIRS in combination with a 5 min ischemic reperfusion technique, both before and at 30 min intervals for two hours post-glucose ingestion. Parameters measured included oxidative metabolic rate (via tissue saturation index [TSI]), immediate recovery slope after occlusion release (TSI10), and total recovery magnitude (ΔTSI). Post-glucose ingestion, both genders demonstrated a surge in blood glucose concentrations at every time point compared to baseline (*p* < 0.001, 0.002, 0.009, and 0.039 for males; *p* < 0.001, < 0.001, = 0.002, and 0.017 for females). Baseline tibial metabolic rate, TSI10, and ΔTSI did not significantly differ between males and females (*p* = 0.734, 0.839, and 0.164, respectively), with no discernible temporal effects in any hemodynamic parameters within each gender (*p* = 0.864, 0.308, and 0.399, respectively, for males; *p* = 0.973, 0.453, and 0.137, respectively, for females). We found comparable tibial hemodynamic responses to OGTT between genders. This study demonstrated the utility of NIRS in evaluating tibial hemodynamic responses to glucose ingestion through OGTT, enriching our understanding of the body’s metabolic responses to glucose intake.

## 1. Introduction

The human body’s ability to metabolize glucose, the primary energy source for the majority of our cells, is essential for overall health and function [1]. Abnormal glucose metabolism may lead to various chronic health anomalies, including but not limited to diabetes mellitus [2], cardiovascular disease [3,4], and specific types of cancer [5,6]. The accurate assessment of glucose metabolism and its impact on various tissues is not only essential for the diagnosis and treatment of these diseases but also for understanding and predicting their etiology and progression.

While most research has concentrated on glucose regulation in tissues like skeletal muscle, liver, and adipose tissue, less is comprehended about how glucose metabolism influences the bone [7]. The complex relationship between glucose metabolism and bone health is attracting increasing attention in biomedical research. It is recognized that bone health is not solely linked to structural and mechanical factors but is also closely connected with the body’s energy metabolism, particularly glucose utilization [8]. Diabetes, which is characterized by chronic elevated blood glucose levels, is correlated with a higher risk of fractures [9], emphasizing the importance of glucose metabolism in bone health.

The Oral Glucose Tolerance Test (OGTT), which measures the body’s response to glucose metabolism, is now a standard method for diagnosing diabetes and prediabetes [10]. The ingestion of glucose triggers a cascade of metabolic, endocrine, and vascular processes that can provide important insights into the body’s metabolic health. For instance, changes in blood flow and vascular reactivity in response to glucose ingestion offer important insights into the metabolic–vascular coupling [11]. While the OGTT is primarily used for systemic glucose monitoring, it also generates various physiological responses to specific tissues that extend beyond mere glucose homeostasis. Paldanius et al. and Westberg-Rasmussen et al. have previously studied skeletal responses to OGTT from an endocrinal point of view by measurement of serum bone turnover markers in healthy subjects [12,13]. Others have investigated the changes in bone turnover markers in response to OGTT in the obese population [14]. However, those serum markers reflect the systematic changes in bone homeostasis and lack spatial specificity. In addition, research regarding hemodynamic changes in bone during OGTT is scarce.

To explore these broader physiological responses, researchers have started to combine the OGTT with various monitoring techniques. Among these, near-infrared spectroscopy (NIRS) has shown promise in detecting the microvascular response. NIRS, a non-invasive technique, employs near-infrared light to measure changes in tissue hemodynamics in real time. Employing NIRS during an OGTT can offer unique observations into the body’s hemodynamic responses to glucose ingestion [11,15]. Using NIRS, Soares and colleagues detected a significant shift in skeletal muscle microvascular activity 90 min after glucose ingestion in young healthy subjects, as evidenced by changes in the oxygen saturation trajectory and the area beneath the oxygen saturation curve after a 5 min ischemic occlusion [11]. Moreover, using similar techniques, it was demonstrated that obese subjects exhibit different skeletal muscle microvascular reactions post-glucose ingestion compared to individuals of normal weight [15]. These investigations indicate NIRS’s capability in capturing muscle microvascular changes stimulated by OGTT, thus contributing significantly to the early detection of potential microvascular dysfunction.

Recent studies have demonstrated the utility of using NIRS to assess long-bone hemodynamics under various physiological conditions [16,17,18]. For example, Zhang et al. concluded that the tibia is characterized by a much slower metabolic rate compared to the tibialis anterior muscle, both assessed simultaneously using NIRS and the ischemia reperfusion technique [16]. Draghici et al. showed that 10 min of rowing exercise induced significant changes in tibial hemoglobin content in able-bodied individuals as measured using NIRS, while no changes were seen for individuals with spinal cord injury [19]. These studies confirmed the validity of NIRS to detect hemodynamic changes in long bone, and hence, may offer a portable, affordable, and easy to use alternative means for such purpose compared to traditionally employed PET scans or contrast-enhanced MRI [20,21]. Given the scarcity of research specifically examining how acute glucose ingestion impacts long-bone hemodynamics in humans, this study aims to address this knowledge gap. We seek to assess and characterize the tibial hemodynamic responses to an OGTT, which will not only provide a nuanced understanding of metabolic influences on bone health but also explore potential diagnostic and therapeutic applications for conditions like osteoporosis and diabetes.

Furthermore, previous studies suggest that gender-based differences might exist in cardiovascular responses and glucose metabolism, attributed largely to hormonal variations and other biological factors [22]. Nevertheless, a detailed understanding of these differences remains incomplete, with only limited studies addressing gender-specific responses in hemodynamics following an OGTT. Consequently, our study aims to explore two main areas of interest. Firstly, we intend to use NIRS to assess the tibial hemodynamic response to an OGTT in a cohort of young healthy individuals. The tibial region presents a convenient location for NIRS measurements, enabling critical insights into peripheral bone microvascular function [20]. Secondly, we aim to compare these responses between males and females to identify potential gender differences, thus enriching the existing body of knowledge on gender-related physiological variations.

## 2. Materials and Methods

### 2.1. Participants

Twenty-eight young healthy individuals (14 males), between the ages of 18–28 were recruited for this study. The inclusion criteria were (1) apparently healthy without known cardiovascular disease or diabetes; (2) no family history of cardiovascular disease or diabetes; (3) no known musculoskeletal disorders, and no injury to the leg for the past year; (4) not currently taking any medication that may affect cardiovascular or musculoskeletal health; and (5) not having participated in regular physical exercise more than once a week (total exercise time < 60 min per week). All female participants reported having regular menstrual cycles. This study was conducted in accordance with the Declaration of Helsinki. Participants signed a consent form prior to any data collection.

### 2.2. Experimental Design

This study used a two-arm parallel group design, with the between-group factor being gender, and the within-group factor being test time. All female participants were tested at the luteal phase of their menstrual cycle. All participants were asked to refrain from vigorous physical exercise or alcohol consumption 48 h prior to the test. They were also asked to refrain from drinking caffeinated beverages or coffee on the day of the test.

On the test day, the participant arrived at our temperature-controlled laboratory (~24 °C) at least 3 h after the last meal to avoid the influence of feeding on glucose level fluctuation. After laying supine on a bed for at least 5 min, the tibial hemodynamic response was evaluated using NIRS. The participant then ingested 75 g of glucose mixed with 500 mL of warm water within 5 min [11]. The tibial hemodynamic response was evaluated every 30 min after glucose ingestion for 2 h. Blood glucose level and heart rate were also evaluated along with NIRS testing.

### 2.3. Heart Rate

Heart rate was recorded to assess central hemodynamic response to OGTT. A heart rate monitor (Polar V800, Polar Electro Oy, Kempele, Finland) was used for heart rate recording for all participants prior to each blood glucose assessment.

### 2.4. Blood Glucose

After cleaning the fingertip using an alcohol wipe, capillary blood was collected with a lancet. Blood glucose level was then assessed using a commercially available, portable blood glucose monitor (isens 631-A, Omron Co., Ltd., Wuhan, China) 1–2 min prior to NIRS testing. The unit used for assessment was mmol/L.

### 2.5. NIRS Testing

A continuous wavelength NIRS device (58 × 28 × 6 mm, PortaLite, Artinis Medical Systems B.V., Einsteinweg, The Netherlands) was used to assess the tibial hemodynamic response on the dominant leg. The PortaLite employs 3 light sources and 1 detector which enables separation distances of 3 cm, 3.5 cm, and 4 cm, and provides an estimated penetration depth of 1.5 cm, 1.75 cm, and 2 cm [16]. It uses the modified Beer–Lambert Law to calculate changes in the oxygenated and deoxygenated hemoglobin concentration within the measured tissue. In addition, the NIRS device also uses the spatially resolved spectroscopic (SRS) method to calculate the tissue saturation index (TSI) of the measured tissue. In SRS, the intensity pattern of the light reflected by the tissue is gauged against the light emitter, and the form of this pattern is associated with the absorption coefficient, which enables the calculation of absolute hemoglobin concentrations [23]. However, since continuous wavelength NIRS devices like PortaLite cannot measure the actual path length, it assumes a steady light scattering coefficient and that the measured tissue is homogeneous [23]. The SRS systems emphasize the contribution of deeper tissues while reducing the contribution of superficial tissues, thus minimizing the influence of skin and adipose tissue on the signal [24]. Therefore, further calculations for hemodynamic changes were done using TSI.

The placement of the NIRS probe was at the level of mid-tibia, measured as halfway between the tibial plateau and the medial malleolus [16]. Care was taken so that the probe was placed directly on top of the tibia thus minimizing the influence of other surrounding tissue. The probe was secured on the skin using double-sided tapes and self-adhesive wraps. A tourniquet was placed right above the knee and was connected to a custom-made rapid cuff inflation system which can inflate the tourniquet to a pre-determined pressure and deflate within 0.5 s. The purpose of this setup was to provide lower limb ischemia in order to assess tibial hemodynamic response [20].

The participant lay on a bed for at least 5 min before the test starts. A 1–2 min baseline was collected, and the tourniquet was rapidly inflated to ~250 mmHg using the rapid cuff occlusion system. The inflation was kept on for 5 min, and the tourniquet was then rapidly deflated. NIRS signal was continuously monitored throughout the test and for at least 8 min after tourniquet release. The NIRS testing was repeated every 30 min after the participant ingested 75 g glucose for 2 h [11,15].

### 2.6. Data Analysis

NIRS data were processed using a custom-written MATLAB script (version 2020b, The Mathworks, Natick, MA, USA). The slope of TSI decrease during ischemic occlusion from 120 s to 150 s (a 30 s window) was calculated to represent tibial oxidative metabolic rate. The first 10 s slope of TSI increase following tourniquet release was calculated to represent tibial microvascular reactivity. The total recovery magnitude of TSI (ΔTSI) was also calculated to indicate the level of reactive hyperemia. These parameters were chosen in accordance with previous studies that measured skeletal muscle microvascular hemodynamic changes using NIRS with these parameters [11,25,26].

The glucose response area under the curve (AUC) was calculated based on Tai’s mathematical model [27].

### 2.7. Statistics

Because there are no studies that we are aware of that specifically examined bone hemodynamic response to glucose ingestion using NIRS, the study sample size was determined based on studies that investigated skeletal muscle microvascular responses to glucose ingestion using NIRS, which generally employed 13 to 16 participants in each group [11,15]. Given a type I error rate of 5% and an estimated effect size of 0.25, this sample size yielded a power of 92.4% [28]. Data were processed using SPSS 27.0 software (IBM Corp., Armonk, NY, USA). Test for normally was carried out for all data using the Shapiro–Wilk method. If the data followed a normal distribution, they were analyzed using two-way ANOVA with time (baseline; 30, 60, 90, and 120 after glucose ingestion) being the within-group factor, and gender (male vs. female) being the between-group factor. For data that do not follow a normal distribution, nonparametric tests were carried out (including independent samples and related samples) to determine if between groups difference and within groups difference exist. The significance level was set at *p* < 0.05. Cohen’s d was calculated whenever applicable to indicate the effect size of the comparisons, with 0.2, 0.5, and 0.8 indicating small, medium, and large effect sizes, respectively [29].

## 3. Results

**Figure 1:**
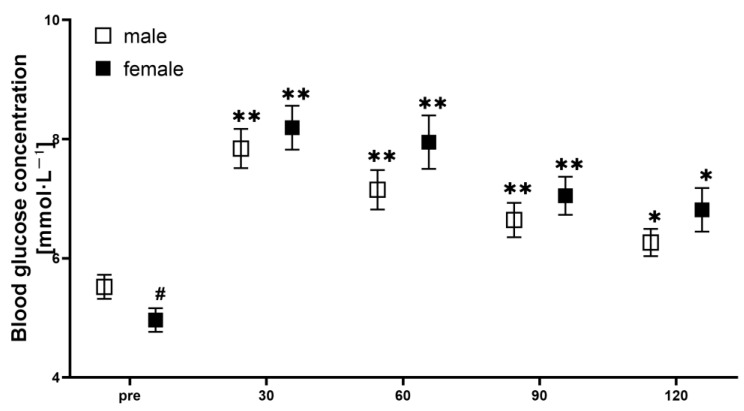
Blood glucose concentration changes in male and female participants throughout the experiment. # significant between groups difference; * *p* < 0.05, ** *p* < 0.01 compared to baseline.

The physical characteristics of all participants are summarized in Table 1. Blood glucose concentration was elevated for males at all post-ingestion time points after glucose ingestion compared to baseline (*p* < 0.001, 0.002, 0.009, and 0.039; d = 2.355, 1.646, 1.249, 0.955, respectively for 30, 60, 90, and 120 min). Similarly, blood glucose concentration was also elevated at all post-ingestion time points for females compared to baseline (*p* < 0.001, < 0.001, 0.002, and = 0.017; d = 3.029, 2.388, 2.171, and 1.746, respectively, for 30, 60, 90, and 120 min; Figure 1). Blood glucose AUC during the OGTT was similar between males and females (826 mmol/120 min vs. 872 mmol/120 min, *p* = 0.267; d = 0.428). HR response to OGTT was unchanged in both groups, as indicated by a lack of interaction effect (*p* = 0.425) as well as main effect (*p* = 0.287 and 0.629 for time and group, respectively).

**Figure 2:**
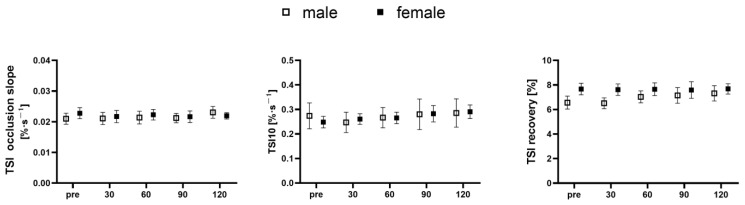
Tibial hemodynamic response to oral glucose tolerance test in male and female participants. TSI, tissue saturation index; TSI10, TSI slope measured 10 s after occlusion release; TSI recovery, TSI total magnitude of recovery after occlusion release.

Tibial oxidative metabolic rate, as assessed using TSI downward slope during occlusion, was similar between males and females at baseline (*p* = 0.734; d = 0.273). No time effect was detected in either male or female participants (*p* = 0.868 and 0.973, respectively). The effect sizes were small for males (d = 0.010, 0.051, 0.035, 0.307, respectively, for 30, 60, 90, and 120 min) and females (d = 0.156, 0.078, 0.171, 0.161, respectively, for 30, 60, 90, and 120 min) compared to their respective baselines Figure 2.

For measurements taken at the reactive hyperemia phase, TSI10 and ΔTSI were similar between male and female participants at baseline (*p* = 0.839, 0.164; d = 0.173, 0.611, respectively), indicating a lack of between-gender variation at rest. No time effect was detected for TSI10 in males and females (*p* = 0.308 and 0.453, respectively, for males and females), and the effect sizes were small for both males (d = 0.155, 0.042, 0.031, and 0.059, respectively, for 30, 60, 90, and 120 min) and females (d = 0.154, 0.203, 0.330, and 0.456, respectively, for 30, 60, 90, and 120 min) compared to their respective baselines. Similarly, no time effect was detected for ΔTSI in males and females (*p* = 0.399 and 0.137, respectively, for males and females). The effect sizes for ΔTSI were small for both males (d = 0.029, 0.256, 0.277, 0.363, respectively, for 30, 60, 90, and 120 min) and females (d = 0.032, 0.010, 0.039, 0.006, respectively for 30, 60, 90, and 120 min) compared to their respective baselines.

## 4. Discussion

To the best of our knowledge, this study represents the first exploration of the tibial hemodynamic response to an OGTT, assessed by NIRS, in healthy young male and female participants. The parameters tested were oxidative metabolic rate during the 5 min occlusion, and microvascular reactivity, measured as the 10 s slope of TSI as well as the total magnitude of recovery of TSI after occlusion release. Contrary to findings from previous studies on skeletal muscle [11,15], the present work indicates that there were no changes in these parameters for tibia during the two hours following OGTT administration. Furthermore, no gender differences were detected in these parameters.

This study is of importance in that it assessed tibial hemodynamic response to an OGTT in humans using NIRS, a subject that has not been previously explored. Gaining insights into how long bones react to glucose intake could enhance our understanding of the intricate relationship between glucose metabolism and the health of bones. The results may offer insights into both clinical as well as future research relevance. As people age, osteoporosis and metabolic diseases like diabetes often co-occur, and understanding the nuanced relationship between bone hemodynamics and glucose metabolism could pave the way for developing integrated approaches to managing these chronic conditions. On the other hand, understanding the distinct responses of bone to glucose could guide the development of more tailored treatments, optimizing outcomes by taking individual physiological responses into account. This study introduces important methodological contributions by applying NIRS technology to study bone metabolism in response to systemic glucose fluctuation. The findings not only enrich our understanding of tissue-specific metabolic responses but also open avenues for future research aimed at developing more personalized and effective treatment regimens for metabolic diseases and bone disorders.

The OGTT is a widely employed method to assess an individual’s capacity to metabolize glucose, thereby determining insulin sensitivity and pancreatic function. By pairing this test with NIRS, a method that can effectively measure tibial hemodynamics and metabolic rate, it is possible to explore the metabolic changes in the tibia and its microvascular response to the metabolic challenge instigated by the OGTT. Contrary to previous studies that found changes in microvascular reactivity for the skeletal muscle 90 min after glucose ingestion [11], no change was observed in the tibia in the current study. The absence of a significant change in tibial hemodynamic response to the OGTT could be attributed to several factors. Firstly, previous investigations on bone metabolism have associated decreased bone formation markers with glucose loading [13,30]. Among those suppressed markers, osteocalcin (OC) and undercarboxylated osteocalcin (ucOC) are recognized as an effector in glucose metabolism [31,32]. Serum levels of OC and ucOC are positively correlated with the results of OGTT [13,30]. The decreased metabolism of osteoblasts can balance out the net effects and result in an undetectable tibial hemodynamic response. Secondly, it is worth noting that the OGTT is a systemic physiological test, and the majority of glucose uptake is regulated by insulin [33]. Importantly, the primary glucose transporter in osteoblasts is GLUT1, which is not insulin dependent [34], suggesting that glucose metabolism in bone is partially unresponsive to changes in blood glucose levels. As such, it may not exhibit the same range of hemodynamic or metabolic changes seen in other tissues following a glucose challenge, rendering it impossible for the current assessment method to capture the nuance changes. Another potential explanation for these findings could be the low metabolic rate of the tibia. Unlike the medial gastrocnemius muscle, whose metabolic rate is known to increase with exercise [16], the tibia’s metabolic rate remains unaffected by exercise. The low metabolic rate renders it impossible to detect changes elicited by OGTT. In addition, another factor to consider relates to the physiological characteristics of the subjects. In this study, we focused on healthy young males and females. Given their likely efficient metabolic responses and hemodynamic regulation, detectable differences in the chosen parameters might not be evident. Prior studies have demonstrated the existence of age-related differences in hemodynamics and metabolic processes [35,36]. These differences might not be present or observable in young, healthy individuals, given their generally more robust and efficient physiological responses.

The absence of gender-based differences in the response to OGTT, as assessed using NIRS, is an intriguing outcome of our experiment. When considering physiological response to stimuli, it is important to consider the influence of hormonal differences between males and females. The crucial role of hormones in metabolism and vascular function is well-established [37,38]. Testosterone, prevalent in males, also influences glucose metabolism and vascular function [39,40]. However, in healthy young individuals, these hormonal differences may not necessarily manifest as observable differences in the tibial hemodynamic response to an OGTT. We must also consider that sex hormone levels fluctuate across the menstrual cycle in women [31], which might have introduced a level of variability that obscured any potential gender-based differences, despite women from the same stage of the menstrual cycle being tested. Examining the impact of hormonal fluctuations on tibial hemodynamic response in future investigations may provide valuable insights into gender-related differences, which is lacking in the current studies and needs in-depth examination. Furthermore, as previously mentioned, the robust metabolic health of our young, healthy cohort might have contributed to the absence of gender differences. Both young males and females tend to have robust metabolic health characterized by efficient glucose metabolism and insulin sensitivity [41]. This efficiency can potentially mute the effects of a metabolic stressor such as an OGTT, leading to homogenous results across genders.

There are methodological considerations for this study. We used TSI as the parameter to measure metabolic rate and microvascular reactivity. As many studies have pointed out, the NIRS signal is influenced by adipose tissue thickness [16,42], which could potentially be problematic when measuring tissues with low metabolic rates, such as bone. However, TSI uses the spatially resolved spectroscopy method to calculate tissue oxygenation, which in theory minimizes the influence of superficial skin and adipose tissue on the signal, and therefore should be more accurate than other NIRS parameters that use the modified Beer–Lambert Law to calculate tissue [24]. On the other hand, we attempted to measure the reactive hyperemia response following occlusion release by calculating the area under the curve for TSI, as was previously done for skeletal muscle [11]. However, there were large inter-individual variabilities, and some participants showed a negative hyperemic response, meaning their post-occlusive TSI may not reach baseline level, making it impossible to compare this parameter. The exact cause for such phenomenon is unclear, but it is possible that due to the slow turnover nature of the bone [16], the hyperemic response may extend beyond the 8 min post-occlusive time that we measured. Future studies with longer post-occlusive monitoring time should be conducted to verify this inference.

The current study’s limitations warrant consideration. First, our study focused on young healthy individuals, excluding individuals with pre-existing vascular conditions or other comorbidities. It is plausible that tibial hemodynamic response to OGTT may emerge under pathological or disease conditions, where underlying physiological differences become more pronounced. Future investigations examining individuals with vascular diseases or conditions characterized by altered metabolic function may shed light on tibial hemodynamic response. Second, it is unclear whether tibial hemodynamic response may be different at different stages of the menstrual cycle in female participants, which should be evaluated in future relevant studies. Third, this study did not measure insulin response to OGTT. Future studies should incorporate insulin measurement as part of the protocol to provide more insights into the observed hemodynamic response. Fourth, although mentioned in the discussion, a direct comparison between bone and muscle response to OGTT could offer more physiological insights, particularly given the fact that the primary glucose transporter in osteoblasts is GLUT1, which is not insulin dependent, while the primary transporter in skeletal muscle is GLUT4, which is insulin dependent. Further investigation should consider evaluating both tissues together, thus enriching our knowledge of how these tissues differentially contribute to glucose metabolism.

## 5. Conclusions

In this study, we observed a lack of general response to OGTT in the tibia, and no gender differences in our cohort of young, healthy individuals. However, these results should be interpreted with the understanding that tissue-specific metabolic properties and methodological aspects can all influence these outcomes. Future research could probe these parameters across different tissues, varied time frames, or within larger, more diverse populations to more fully elucidate the nuanced effects of gender on hemodynamic responses to a glucose challenge.

## Figures and Tables

**Table 1:** Physical characteristics for all participants.

	Total	Males	Females	*p*
N	28	14	14	-
Age (years)	22.1 (2.6)	22.3 (2.9)	22.0 (2.4)	0.839
Height, cm	166.5 (7.9)	172.5 (4.4)	160.5 (5.7)	<0.001
Weight, kg	60.0 (12.4)	67.6 (10.9)	52.3 (8.4)	<0.001
BMI, kg/m^2^	21.5 (3.7)	22.8 (4.1)	20.2 (2.8)	0.077

## Data Availability

Data pertinent to this study are available via contacting the corresponding author.
